# Consistency between Self-Reported and Recorded Values for Clinical Measures

**DOI:** 10.1155/2016/4364761

**Published:** 2016-01-31

**Authors:** Joseph Thomas III, Mindy Paulet, Jigar R. Rajpura

**Affiliations:** ^1^Purdue University, College of Pharmacy and Regenstrief Center for Healthcare Engineering, Center for Health Outcomes Research and Policy, Robert Heine Pharmacy Building, Room 502A, 575 Stadium Mall Drive, West Lafayette, IN 47907-2091, USA; ^2^WorkLife Programs Human Resources Purdue University, 1601 West State Street, West Lafayette, IN 47907-2091, USA; ^3^Purdue University, College of Pharmacy and Regenstrief Center for Healthcare Engineering, Center for Health Outcomes Research and Policy, Robert Heine Pharmacy Building, Room 515, 575 Stadium Mall Drive, West Lafayette, IN 47907-2091, USA

## Abstract

*Objectives*. This study evaluated consistency between self-reported values for clinical measures and recorded clinical measures.* Methods*. Self-reported values were collected for the clinical measures: systolic blood pressure, diastolic blood pressure, glucose level, height, weight, and cholesterol from health risk assessments completed by enrollees in a privately insured cohort. Body mass index (BMI) was computed from reported height and weight. Practitioner recorded values for the clinical measures were obtained from health screenings. We used bivariate Pearson correlation analysis and descriptive statistics to evaluate consistency between self-reported data and recorded clinic measurements.* Results*. There was high correlation between self-reported clinical values and recorded clinical measures for diastolic blood pressure (*r* = 0.91, *P* = <0.0001), systolic blood pressure (*r* = 0.93, *P* = <0.0001), cholesterol (*r* = 0.97, *P* = <0.0001), body mass index (*r* = 0.96, *P* = <0.0001), glucose (*r* = 0.96, *P* = <0.0001), weight (*r* = 0.98, *P* = <0.0001), and height (*r* = 0.89, *P* = <0.0001).* Conclusions*. Self-reported clinical values for each of the eight clinical measures examined had good consistency with practitioner recorded data.

## 1. Introduction

Self-reported clinical measures are inexpensive and take less time to collect [1–3] than individual clinic examinations in large study samples [4–8]. Consequently, use of self-reported clinical values collected from self-administered questionnaires as a source of information on health status is commonplace [5, 9–11].

A number of studies have examined consistency between self-reported and recorded values for clinical measures, but the studies have often been limited to a single measure [12–19]. Findings from the prior studies also vary in the level of consistency reported [14, 18, 20–23]. Additional data on consistency of self-reported data with clinically recorded data would be useful in guiding use of self-reported data in research and in management of patients.

Pieper et al. [18] conducted three surveys among adults aged 25 to 74 years from 1980 to 1982 (*N* = 4086), 1985 to 1987 (*N* = 5735), and 1990 to 1992 (*N* = 6305) in the Minneapolis-St. Paul, Minnesota, metropolitan area as part of the Minnesota Heart Survey. Participants completed a short interview in their homes, which included questions about cholesterol level, blood pressure, cigarette smoking, prescription medication use, education, and occupation. Individuals' total serum cholesterol levels were measured at the first clinic visit after completion of the interview. Among participants who provided a value for their total serum cholesterol level, the correlation between self-reported total serum cholesterol and clinically recorded total serum cholesterol ranged from 0.54 to 0.75 across all study periods.

Huang et al. [14] conducted a randomized double blind clinical trial of low-dose aspirin and vitamin E in primary prevention of cardiovascular disease and cancer in a sample of 39,876 apparently healthy women aged 45 years or older from registries of health professionals between 1993 and 1996. During enrollment, participants were asked to report their total serum cholesterol levels as a part of baseline questionnaires. Following the questionnaire, participants willing to provide a venous blood sample were mailed blood collection kits. The median time between the date of blood collection and the date of self-reported total serum cholesterol was 37 days (interquartile range, 4 to 82 days). Huang et al. reported a correlation of 0.54 between self-reported and measured total cholesterol levels. Huang et al. also reported that in general the study sample underestimated total cholesterol by 9.7 mg/dL.

Body mass index (BMI) is often used as an indicator of overweight and obesity and obesity has been identified as an important risk factor for many diseases, including cardiovascular disease and diabetes [24, 25]. Since BMI is computed based on height and weight, a number of studies have examined consistency between self-reported height and weight with recorded values and how differences affect estimated BMI values.

Fonseca et al. [21] recruited 462 students in grades 6, 8, and 10 from 12 Portuguese public schools to assess accuracy of adolescent's reported weight and height in comparison with recorded weight and height. All students were asked to answer a one-sheet anonymous questionnaire in which they reported their weight and height. Investigators computed student's body mass index (BMI) from the self-reported height and weight. After students completed the survey, height and weight of students were measured and BMI computed based on the measurements. Fonseca et al. found high correlation between reported and recorded weight (0.84) and between reported and recorded height (0.96). Among girls, self-report based BMI and measurement based BMI were highly correlated, with reported BMI explaining approximately 79% (*R* ^2^ = 0.785) of the variance in measured BMI, with a standard error of approximately 1.6 kg/m^2^. Among boys, computed BMI (from self-reported height and weight) explained 65% (*R* ^2^ = 0.651) of the variance in measurement based BMI, with a standard error of 2.1 kg/m^2^.

Niedhammer et al. [23] studied 20,624 men aged 40 to 50 years and women aged 35 to 50 years, working in a French national company in 2000 to examine accuracy of self-reported weight and height and resulting body mass index (BMI) in comparison with clinically measured BMI. Self-reported weight and height of individuals in the study cohort were collected in an annual medical questionnaire. Recorded clinical measures were obtained from a follow-up annual medical examination in the same year. For each subject, physician measured height and weight data were matched with self-reported data from the self-administered questionnaire nearest in time.

Subjects were excluded from the analysis if the interval between the measured and self-reported data was longer than 6 months. Correlation between self-reported and measured weight was 0.98 and correlation between self-reported and measured height was 0.98. Correlation between self-report based BMI and BMI based on recorded measures was 0.97. The mean difference between measured and self-reported weight was greater for women (0.85 kg) than men (0.54 kg) and there was a greater mean difference between measured and self-reported BMI for women (0.44 kg/m^2^) than men (0.29 kg/m^2^).

McAdams et al. [22] analyzed data from National Health and Nutrition Education Study III participants 20 years old or older to examine accuracy of self-reported weight and height and the resulting body mass index (BMI) in comparison with clinically measured BMI. Investigators collected self-reported information from a home based self-administered questionnaire. That was followed by a home based clinical examination conducted by trained health technicians.

Average time interval between collection of reported data and the clinical examination was 33.5 days. McAdams found study participants under reported weight by 0.56 kg and height by 0.76 cm. Subsequently self-reported BMI (25.07 kg/m^2^) was lower than measured BMI (25.52 kg/m^2^). However, the correlations between self-reported and measured BMI values were high (0.95 for whites, 0.93 for blacks, and 0.90 for Mexican Americans).

Brener et al. [20] recruited 4619 students in grades 9 to 12. Students completed questionnaires to report their height and weight. Following completion of self-administered questionnaires, students' height and weight were measured and recorded. Average time between collection of student reported data and the clinical examinations was 3 days. Brener reported strong overall correlations between self-reported height (0.90), weight (0.93), and BMI (0.89) with their measured values. Students tended to overreport their height by 2.7 inches and to underreport their weight by 3.5 pounds. Consequently, self-reported BMI values were an average of 2.6 kg/m^2^ lower than BMI based on recorded values.

We found no studies that assessed consistency of self-reported blood glucose level or self-reported blood pressure measures with recorded clinical measures.

The objective of this study was to evaluate concordance between self-reported blood pressure, cholesterol, blood glucose, height, weight, and BMI with recorded clinical measures.

## 2. Methods

### 2.1. Sample Selection

The cross-sectional study sample consisted of individuals 18 years or older as of January 2006 who were enrolled in a privately insured cohort employed by a large state university. Individuals eligible for participation included all covered employees who were hired on or before December 1 of the year prior to each health risk assessment. Participation was limited to faculty or staff classified as “benefit eligible,” that is, those who were employed half-time or more and their spouses or same sex domestic partners, and was voluntary. Individuals who had completed health risk assessments were eligible for inclusion in the study sample. Recorded clinical measures were obtained from health care screening prior to or subsequent to completion of health risk assessments. The project was reviewed and approved by the Purdue University Institutional Review Board, West Lafayette, Indiana.

### 2.2. Data Sources and Study Variables

Data on self-reported clinical measures were taken from health risk assessment surveys completed by privately insured employees of a state university. Data on clinically recorded values for the clinical measures were taken from data recorded during health screenings. The self-reported data and the clinically recorded data included variables for systolic blood pressure, diastolic blood pressure, blood cholesterol, blood glucose, weight in pounds, and height in inches. BMI values were computed from individual's reported height and weight measures and from clinically recorded height and weight. Information on demographic variables, including age, gender, staff type, income, and whether the person completing the assessment was an employee or spouse covered under the employee's insurance plan, was obtained from plan enrollment files and was based on January of 2006 or the earliest enrollment month for each individual during that year.

To include only plausible clinical values ranges were established for each clinical measure at which values below or above the specified range were excluded from the analysis. The intervals established for each clinical measure were systolic and diastolic BP was between 40 mm/Hg and 250 mm/Hg, total serum cholesterol levels between 75 mg/dL and 500 mg/dL, body mass index between 10 kg/m^2^ and 100 kg/m^2^, serum glucose levels between 10 mg/dL and 500 mg/dL, weight between 60 lbs and 700 lbs, and height between 36 and 86 inches.

### 2.3. Statistical Analysis

The study sample was categorized based on demographic characteristics including age, gender, staff type, employee/spouse, and income. Pearson's correlation analysis was used to assess consistency between self-reported clinical values and recorded clinic measurements. Mean differences, standard deviations, and absolute differences between self-reported and recorded clinic measures were calculated. All analyses were conducted using SAS version 9.1 (SAS Institute, Cary, NC, USA) with 0.05 alpha level.

## 3. Results

As shown in Table 1 a total of 12,752 respondents met sample inclusion criteria of being at least 18 years of age. Out of those, 3630 respondents with a clinical examination within ±90 days of their Health risk assessment were selected for further analyses. More than half (58.8%) of the sample was female. Only a small number values had to be excluded for being outside the established range for a measure. The highest number for any value was 21 values, and most, 16, of those were recorded values that were excluded. None of the recorded or self-reported diastolic blood pressure had to be excluded for being above or below the established range. One recorded systolic blood pressure value was excluded for being below the established range and no self-reported systolic blood pressures were excluded for being above or below the established range. Two recorded cholesterol values were excluded for being below the established range but none of the self-reported cholesterol values had to be excluded for being outside the range. For recorded glucose, values were excluded for being below the range while six self-reported glucose values were excluded for being outside the established range, five for being below the range and one for being over the range. Three recorded weight values were excluded for being below the range and no self-reported weight values were outside the range. A total of 16 height values were excluded for being outside the range, seven for being below the range, and nine for being over the range. Only five self-reported height values were excluded for being outside the range and all five were above the range.

Table 2 shows mean differences between self-reported values and recorded clinical measures and correlations between self-reported clinical values and recorded clinic measures for diastolic blood pressure, systolic blood pressure, cholesterol, BMI, glucose, weight, and height. Absolute mean differences were also examined since values above and below the recorded values might tend to cancel each other out in mean differences. As seen in Table 2, the mean differences self-reported and recorded values were low. The mean absolute differences between the values were also low. Correlations were high and ranged from 0.89 for height to 0.98 for weight.

In assessing differences between female and male respondents in Table 3 with respect to reporting accuracy across clinical measures only weight, height, and BMI were observed to differ significantly in level of consistency across gender. Males were observed to overreport their weight by 2.26 lbs and females by 1.02 lbs. Subsequently BMI was overreported across both gender groups. In Table 4, absolute difference between self-reported weight and recorded weight measure was observed to be 2.12 pounds for females and 3.19 pounds for males.

## 4. Discussion

Prior studies of consistency between self-reported clinical measures and recorded values have often been limited to single clinical measures or subgroups. We found no studies that evaluated concordance between self-reported blood pressure or self-reported blood glucose levels with prior clinically measured data.

Similar to the findings in studies conducted by Pieper et al. [18], Niedhammer et al. [23], McAdams et al. [22], Brener et al. [20], Huang et al. [14], Newell et al. [2], and Harlow and Linet [5] in large representative cohorts, we observed that self-reported clinical values are consistent with recorded clinic measures. Also, similar to results observed by Pieper et al. [18], Huang et al. [14], Niedhammer et al. [23], McAdams et al. [22], and Brener et al. [20], we found strong positive correlations and low differences between reported and recorded clinic measures. In Huang et al.'s [14] study on accuracy of self-reported cholesterol in women, 37.8% of the study sample overreported their cholesterol [14]. Similarly, in our study, respondents overreported their cholesterol on average by only 0.10 mg/dL, which is of little clinical significance. These findings collectively suggest that information collected through administered health survey in our privately insured health cohort is accurate.

*Limitations*. The study sample consisted of individuals only from a small geographical region and a specific private cohort that require caution in generalizing the findings.

## 5. Conclusion

In conclusion, self-reported clinical values could be reliable in cohort based epidemiologic studies for blood pressure, cholesterol, glucose, BMI, and weight measures in a private insurance-based cohort. Self-administered health survey continues to be important tools in epidemiology research.

## Figures and Tables

**Table 1:** Sample Distribution by Demographic Characteristics.

Characteristics	Total *N* = 12,752		Individuals with health risk assessment and no health screen within 90 days of risk assessment *N* = 9,122		Individuals with health risk assessment and a health screen within 90 days of risk assessment *N* = 3,630		*P* value^1^
	Number	%	Number	%	Number	%	
Age							
Less than 30	1,274	9.9	865	9.4	409	11.2	<0.001
30 to 39	2,771	21.7	1,939	21.2	832	22.9	
40 to 49	3,285	25.7	2,305	25.2	980	26.9	
50 to 59	3,795	29.7	2,750	30.1	1,045	28.7	
60 to 69	1,481	11.6	1,134	12.4	347	9.5	
Gender							
Female	7,306	57.2	5,171	56.6	2,135	58.5	0.028
Male	5,446	42.7	3,951	43.3	1,495	41.8	
Staff type							
Administrative	4,662	36.5	3,189	34.9	1,473	40.5	<0.001
Clerical	2,121	16.6	1,541	16.9	580	15.9	
Faculty	2,807	22.0	2,286	25.0	521	14.3	
Operations	682	5.3	497	5.4	185	5.1	
Service	2,479	19.4	1,608	17.6	871	23.9	
Employee/spouse							
Employee	9,645	75.6	6,105	65.9	3,630	100.0	<0.001
Spouse	3,106	24.3	3,106	34.0	0	0	
Income							
Less than $30,000	4,479	35.1	3,278	35.9	1,201	33.0	<0.001
$30,000 to $60,000	5,374	41.9	3,608	39.5	1,325	47.9	
$60,000 to $90,000	1,784	13.9	1,349	14.7	435	13.9	
$90,000 to $120,000	718	5.6	559	6.1	159	4.3	
$120,000 to $150,000	47	0.2	37	0.4	10	0.2	
$150,000 to 180,000	102	0.8	80	0.8	22	0.6	
More than $180,000	63	0.4	50	0.5	13	0.3	

^1^ *P* values from chi-square test comparing distributions on each demographic variable between individuals who completed health risk assessments and those who did health risk assessments and had screenings with 90 days of the health risk assessment.

**Table 2:** Mean differences between self-reported values and recorded values on clinical measures.

Clinical measures	Number	Mean difference	Standard deviation	*P* value^1^	Absolute difference	Standard deviation	*P* value^1^	Pearson corr.	*P* value
Diastolic BP	3,612	0.86	4.10	<0.001	1.63	3.86	<0.001	0.91	<0.001
Systolic BP	3,611	0.99	4.86	<0.001	1.82	4.62	<0.001	0.93	<0.001
Cholesterol	3,597	0.10	10.58	0.576	1.54	10.47	<0.001	0.97	<0.001
BMI	3,357	0.71	1.80	<0.001	1.05	1.63	<0.001	0.96	<0.001
Glucose	3,590	0.39	8.66	0.007	1.31	8.57	<0.001	0.96	<0.001
Weight	3,607	1.53	8.44	<0.001	2.56	8.18	<0.001	0.98	<0.001
Height	3,593	0.03	1.93	0.382	0.76	1.78	<0.001	0.89	<0.001

^1^ *P* values from paired *t*-test between values from self-reported and recorded clinical measures.

**Table 3:** Comparison of difference between male and female participants' self-reported values and recorded values on clinical measures.

Clinical measures	Number	Females		Number	Males		*P* value^1^
		Mean difference	Standard deviation		Mean difference	Standard deviation	
Diastolic BP	2,123	0.83	4.02	1,488	0.90	4.22	0.630
Systolic BP	2,122	0.96	4.73	1,488	1.02	5.04	0.720
Cholesterol	2,121	−0.04	11.08	1,475	0.30	9.82	0.328
BMI	1,999	0.61	1.69	1,357	0.85	1.95	0.002
Glucose	2,117	0.35	8.54	1,472	0.44	8.83	0.774
Weight	2,119	1.02	7.88	1,487	2.26	9.12	<0.001
Height	2,115	0.08	1.87	1,477	−0.05	2.01	0.039

^1^ *P* values from *t*-test comparing mean differences in self-reported and recorded clinical measures between male and female participants.

**Table 4:** Comparison of absolute difference between male and female participants' self-reported values and recorded values on clinical measures.

Clinical measures	Number	Females		Number	Males		*P* value^1^
		Mean difference	Standard deviation		Mean difference	Standard deviation	
Diastolic BP	2,123	1.54	3.81	1,488	1.75	3.94	0.103
Systolic BP	2,122	1.73	4.51	1,488	1.93	4.77	0.205
Cholesterol	2,121	1.54	10.97	1,475	1.54	9.71	0.989
BMI	1,999	1.00	1.49	1,357	1.13	1.80	0.021
Glucose	2,117	1.23	8.45	1,472	1.41	8.73	0.552
Weight	2,119	2.12	7.66	1,487	3.19	8.84	0.002
Height	2,115	0.75	1.72	1,477	0.76	1.86	0.800

^1^ *P* values from *t*-test comparing mean differences in self-reported and recorded clinical measures between male and female participants.
